# Carbon dioxide utilization in propylene carbonate production process

**DOI:** 10.1038/s41598-024-65115-z

**Published:** 2024-06-22

**Authors:** Erfan Esmaeili-Chelan, Fatemeh Bahadori

**Affiliations:** Faculty of Chemical Engineering, Urmia University of Technology, Urmia, Iran

**Keywords:** Propylene carbonate, Carbon dioxide utilization, Environment, Green process, Chemical engineering, Pollution remediation

## Abstract

Utilizing carbon dioxide in chemical processes is one of the suggested remedies for reducing its atmospheric concentration. In this paper, the simulation of the propylene carbonate production process using 1,2-propanediol and carbon dioxide has been performed. The impact of temperature was examined between 75 and 400°C. It has been found that as temperature rises, reaction conversion increases. Additionally, the impact of recycling ratio was examined in the range of 0.1–0.5, which demonstrates that a rise in the recycle ratio results in a fall in conversion. Furthermore; it was observed that the pressure initially increases the solubility of carbon dioxide in 1,2-propanediol and improves the conversion of the reaction, but when enough carbon dioxide is supplied in the reaction, increasing the pressure does not affect the corresponding reaction. The effect of all studied parameters depends on the residence time of the reactants in the reactor. Investigating the interaction of parameters and optimizing the process has shown several optimal point of the process such as temperature of 295 °C, a recycle ratio 0.1, feed ratio 0.8 and a residence time of 12.61 h, with the related conversion being 59.6%.

## Introduction

Organic carbonates like dimethyl carbonate (DMC), diethyl carbonate (DEC), and propylene carbonate (PC) have received increased attention in recent decades due to their unique features^1,2^. These substances are used to produce complex carbonates or polycarbonates^3^. In reactions such as the manufacture of polyurethane or as replacements for damaging solvents like dichloromethane or toluene, carbonates are appropriate substitutes for the extremely poisonous and dangerous compound phosgene^4^. Both dimethyl carbonate and diethyl carbonate along with glycerol carbonate, are frequently employed as petrol additives and as solvents in lithium-ion batteries^5–7^. Due to its degradability, high boiling and flame temperatures, low odor level, and comparatively low evaporation rate and toxicity, propylene carbonate is frequently employed in the synthesis of different compounds, gas separation, electrochemistry, metal extraction, etc.^8^. Currently, propylene carbonate is also used to produce dimethyl carbonate through transesterification with methanol, which provides a new route for the synthesis of propylene carbonate^9^.

Typically, most carbonates and polycarbonates are synthesized using poisonous chemicals such as phosgene, bisphenol A, and diphenyl carbonate. Another process is the oxidative carbonylation of methanol or methyl nitrile with carbon monoxide. Although this process is very favorable in terms of thermodynamics, its use is limited due to the reaction occurring in the presence of toxic and corrosive chemicals. Additionally, cyclic carbonates are mainly synthesized by adding carbon dioxide to cyclic oxides, which is a dangerous process due to the presence of propylene oxide^10,11^. 1,2-propanediol (propylene glycol), a compound containing two alcohol groups, is extensively considered for propylene carbonate production. 1,2-propanediol can react with urea or carbon dioxide to produce propylene carbonate. Since utilizing carbon dioxide in chemical processes provides the required carbon from a significant greenhouse gas, studying and developing these processes has been prioritized.

During the reaction between carbon dioxide and alcohol or bio-alcohol, dimethyl carbonate, diethyl carbonate, propylene carbonate, along with water are produced. The biocompatibility of these processes has resulted in increasing interest in synthesizing organic carbonates using carbon dioxide and alcohol^12–14^. Carbon dioxide is a low-activity gas and usually reacts at high temperatures. Providing the heat for the reaction typically requires burning fossil fuels, which can emit additional carbon dioxide. Therefore, the usage of carbon dioxide in processes that have low temperatures is more appropriate. Due to the utilization of carbon dioxide in producing propylene carbonate at low temperature; more studies, evaluation, and optimization of affecting parameters in the process is required. So, in this research, the simulation and optimization of propylene carbonate production using carbon dioxide have been studied.

## Simulation procedure

In this section, modeling and simulation of propylene carbonate production using 1,2-propanediol and carbon dioxide have been presented. Simulations of this study have been carried out based on the experiments performed by Décultot et al.^15^. The production of propylene carbonate using 1,2-propanediol and carbon dioxide was performed over a calcinated cerium oxide (IV) catalyst. The reaction occurred in an autoclave with a capacity of 100 ml. During this experiment, first, 50 ml of 1,2-propanediol was filled in the reactor with 2 g of CeO_2_. Forty-five minutes were considered for CO_2_ to dissolve in 1,2-propanediol, and the temperature increased to the reaction take place (Décultot et al.^15^).

The reaction of 1,2-propanediol and carbon dioxide to produce propylene carbonate takes place according to the following reaction^15^:

1 $$C{O_2} + {C_3}{H_8}{O_2}\left( {1,2{\text{-}}propandiol} \right) \leftrightarrow {C_4}{H_6}{O_3}\left( {propylene\;carbonate} \right) + {H_2}O$$

2 $$r_A^{PC} = {k^{PC}}\left( {\frac{{\frac{{\left[ {1,2{\text{PPD}}} \right]{{\text{P}}_{{\text{CO2}}}}}}{{{{\text{C}}_0}{{\text{P}}_0}}} - \frac{{[{\text{PC}}][{{\text{H}}_2}{\text{O}}]}}{{{{\text{K}}_{{\text{eq}}}}{\text{C}}_0^2}}}}{{{{\left( {1 + {\text{K}}_{{\text{A}}1}^{{\text{PC}}}\frac{{{{\text{P}}_{{\text{CO2}}}}}}{{{{\text{P}}_{\text{0}}}}} + {\text{K}}_{{\text{A}}2}^{{\text{PC}}}\frac{{\left[ {1,2{\text{PPD}}} \right]}}{{{{\text{C}}_{\text{0}}}}} + \frac{{[{\text{PC}}]}}{{{{\text{C}}_{\text{0}}}{\text{K}}_{{\text{A}}4}^{{\text{PC}}}}} + \frac{{[{{\text{H}}_2}{\text{O}}]}}{{{{\text{C}}_{\text{0}}}{\text{K}}_{{\text{A}}5}^{{\text{PC}}}}}} \right)}^2}}}} \right)$$

The kinetic parameters of the Eq. (6) have been presented in Table S1.

Where equilibrium constant as a function of enthalpy and entropy is as follows:

3 $$Ln{\text{K}}_{\text{eq}}=\frac{-{\Delta }_{r}{H}_{T}^{0}}{RT}+\frac{{\Delta }_{r}{S}_{T}^{0}}{R}$$

### Process simulation

**Figure 1:**
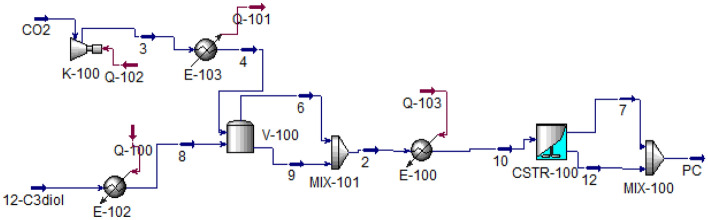
Simulation of propylene carbonate production process.

Simulation of propylene carbonate production using 1,2-propanediol and carbon dioxide has been performed with Aspen HYSYS 10. The NRTL equation of state has been implemented to predict the thermodynamic behavior of process fluids. Figure 1 shows the simulation of the process.

### Verifying the validity of results

**Figure 2:**
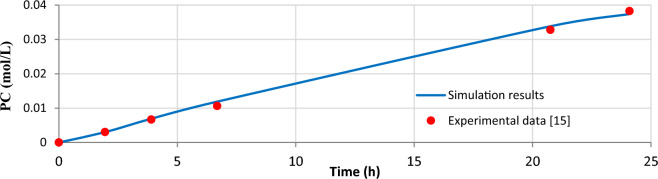
Comparison of experimental data^15^ with simulation results at 103 °C.

Figure 2 shows the effects of residence time on propylene carbonate (PC) production at the temperature of 103 ͦ C based on experimental data reported by Décultot et al.^15^ and simulation results. It is shown that increasing the residence time enhances the PC production rate. Comparing the results showed that the simulation results are in good agreement with experimental data (average error is 3.69%). So, the simulation is developed for further studies.

**Figure 3:**
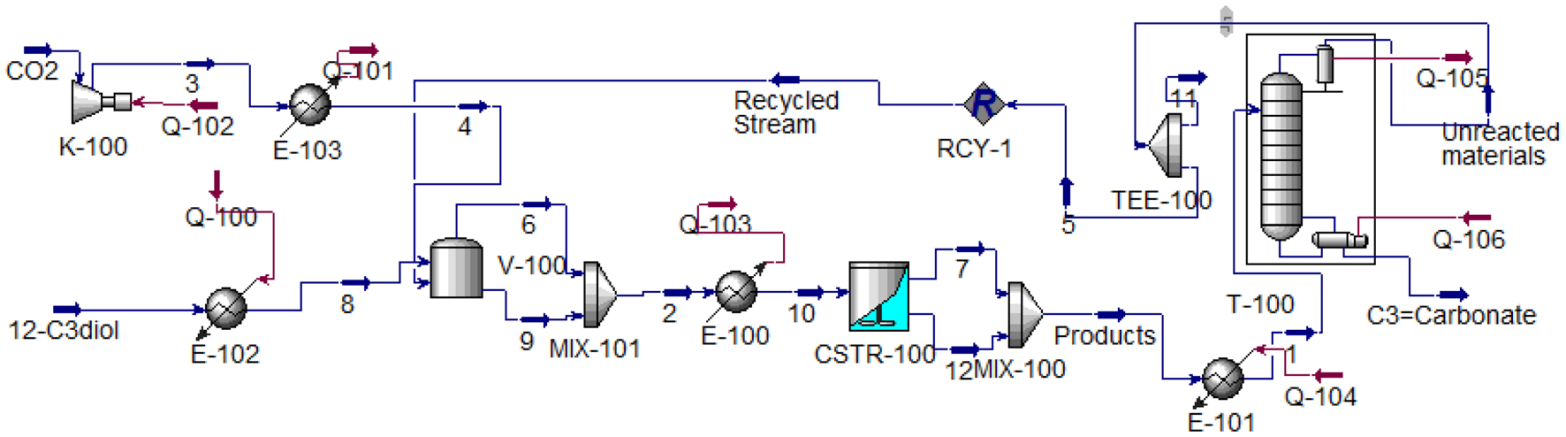
Developed PC production process.

According to verify the validity of the simulation results, the simulation was implemented to develop the process. In the developed simulation, a flash drum and the distillation column have been considered to separate the products and a recycled stream to return part of unreacted raw materials to the reactor. The overview of the process, which includes the reactor and separation section, has been shown in Fig. 3.

### Process optimization

The optimization of the process leads to the achieving of optimal design of the process. In process optimization, an experiment (a simulation in this study) is designed to examine the multiple effects of different variables. Then, a regression model will establish a relationship between the response and the variables. This paper uses Design Expert 12 along with Response Surface Methodology (RSM) to investigate the interaction between variables and optimize the process. RSM is a statistical technique to build a model for optimizing the response (output variable) that is affected by independent variables. Box Behnken Design (BBD) has been used in this paper to optimize the propylene carbonate production process. The significant parameters of the process including temperature, residence time, Feed ratio, and recycle ratio have been examined on 1,2-propanediol conversion (response). A total of thirty-one runs have been carried out in a standard manner. In addition, the Quadratic model has been used for multiple regression analysis. The prediction of the response function has been presented as follows^16^:

4 $$Y= {\beta }_{0}+{\sum }_{j=1}^{k}{\beta }_{j}{X}_{i}+{\sum }_{j=1}^{k}{\beta }_{ii}{X}_{i}^{2}+{\sum }_{i}^{k-1}{\beta }_{ij}{X}_{j}{X}_{i}+e$$

The values for the factor’s level in RSM analysis are: 2 < A: Residence time (hr) < 16; 100 < B: Temperature (°C) < 300; 0.4 < C: Feed Ratio < 0.8; 0.1 < D: Recycle ratio < 0.5.

The Behnken box arrangement for factor and response levels has been shown in Table S2 and Fig. S1.

## Results and discussion

In this section, effective parameters on PC production including temperature, residence time, feed ratio, and recycle ratio have been investigated.

### Temperature effects on PC production

**Figure 4:**
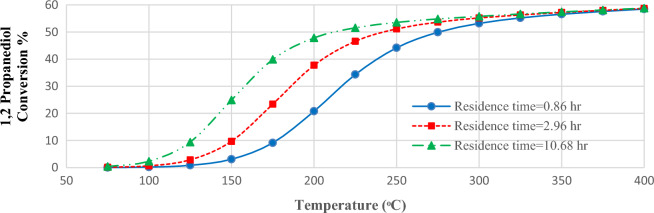
The conversion of 1,2-propanediol versus temperature in 3 different residence times (recycle ratio = 0.1).

Figure 4 shows the effects of feed temperature on PC production in 3 residence times of 0.86, 2.96, and 10.68 h. It is seen that by increasing the temperature, the conversion of reaction increases rapidly and after the temperature of 250 °C the increasing rate lowers, i.e., the 1,2 propanediol conversion in the residence time of 10.68 h at a temperature of 150 °C is 24.91%, while the conversion at a temperature of 250 °C reaches 53.54%. Moreover, it can be seen that the conversion rises by increasing the residence time. In fact, the small kinetic constant needs higher temperatures to provide the activation energy. A similar trend has been reported by Huang et al.^17^.

### Feed ratio effects on PC production

**Figure 5:**
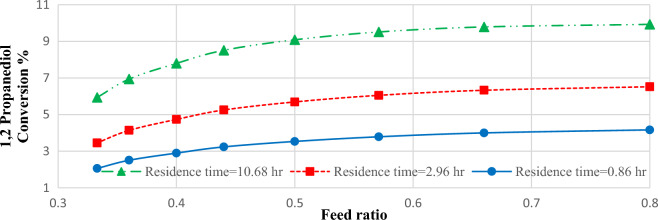
The conversion of 1,2-propanediol versus feed ratio in three different residence times (temperature = 200 °C, and recycle ratio = 0.1).

Figure 5 shows the effects of feed ratio on propylene carbonate production in three residence times of 0.86, 2.96, and 10.68 h. It is shown that variation in feed ratio has a mild effect on 1,2-propanediol conversion and the limiting parameter in feed composition is related to the solubility of carbon dioxide in 1,2-propanediol.

### Recycling effects on PC production

**Figure 6:**
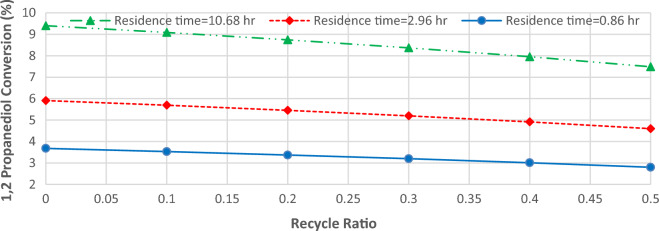
The conversion of 1,2-propanediol versus recycle ratio in three residence times (temperature = 250 °C).

Figure 6 shows the effects of increasing the recycle ratio on the conversion of 1,2-propanediol in residence times of 0.86, 2.96, and 10.68 h. Since by recycling a part of effluent into the reactor, the resident time of reactants decreases, it is evident that by increasing the recycle ratio, the conversion of 1,2-propanediol descends. A similar trend has been reported by Huang et al.^18^.

### Analysis of variance (ANOVA)

Design Expert 12 has been used to evaluate the interactions between key variables in the manufacturing of propylene carbonate in order to achieve the best possible operating conditions. The procedure has been examined using the Quadratic model, sequential Sum of Squares, and natural logarithm transformation. Table S3 presents the results of the 1,2-propanediol conversion analysis of variance (ANOVA) along with the F-value and the P-value. The aforementioned model is significant with the selected parameters when the P-value is less than 0.1 for each of the interactions. The interactions of AB, AC, AD, BC, CD, A^2^, and B^2^ have a considerable effect on the conversion of 1,2-propanediol as shown in Table S3, however; the interactions of BD, C^2^, and D^2^ exceeded 0.1000. Additionally, the model’s F-value, which measures its significance, is 10,323.79. In Table S3, the model’s statistical parameters are also included. The predicted R square shows how well the used regression model can predict the results for new points. The predicted R-square’s best value is 1, hence the value of 0.9999 denotes the model’s accuracy. The adjusted error of 0.9998 also provides a precise measurement of the signal-to- noise ratio. For Adeq precision, a value of less than 4 is preferred for the signal-to-noise ratio. Therefore, this model can be used to improve the process and predict the response:

5 $$\begin{aligned} {ln}(conversion) & =2.39+0.5891\times A+2.01\times B+0.0243\times C \\ & \quad -0.0093\times D-0.1522\times AB+0.0292\times AC \\ & \quad +0.0212*AD +0.0158\times BC-0.0419\times CD \\ & \quad -0.3652\times A^{2}-0.4746\times B^{2}\end{aligned}$$

**Figure 7:**
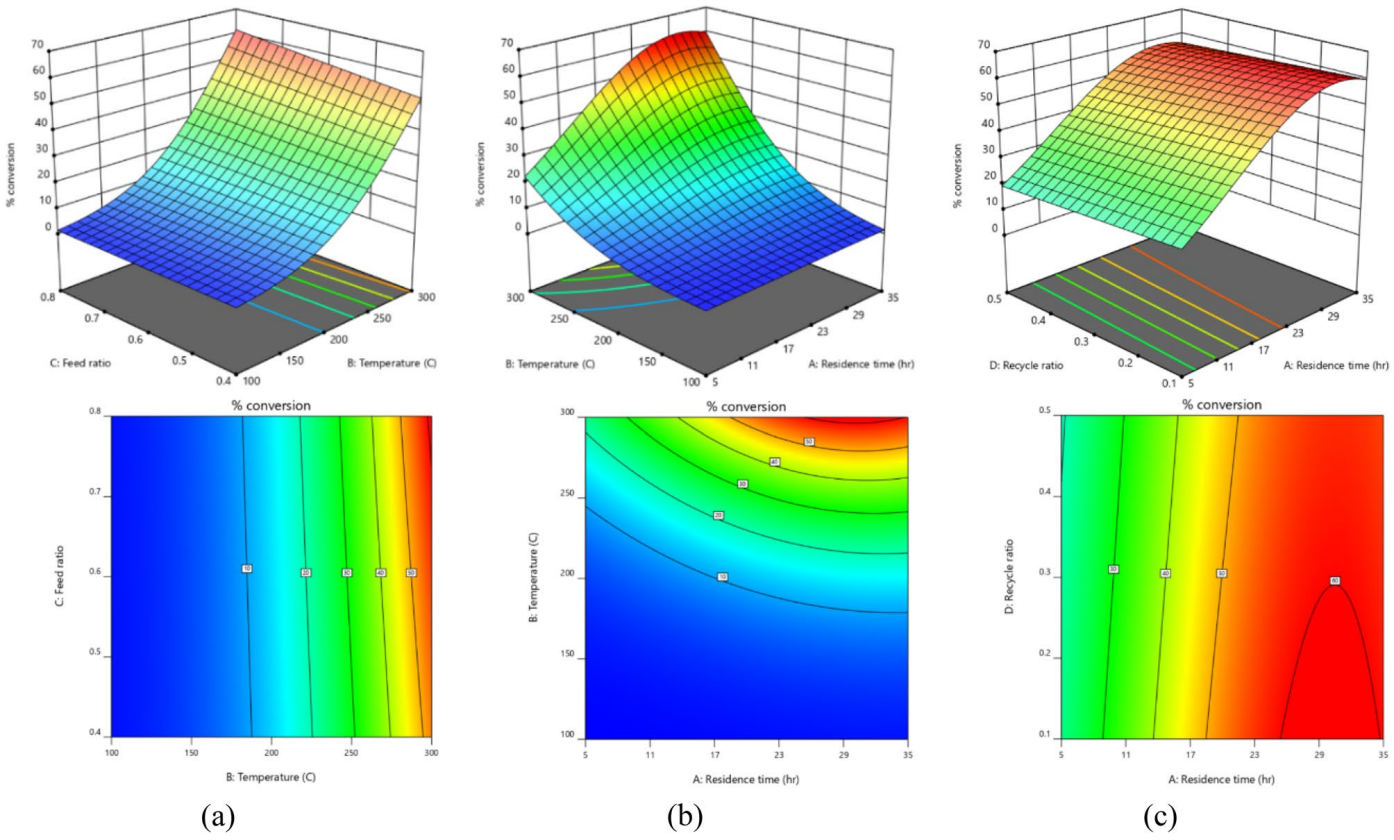
Two and three-dimensional effects of (**a**) temperature-feed ratio on conversion of 1,2-propandion (Residence time = 27.5 h, Recycle ratio = 0.1), (**b**) temperature-residence time on conversion of 1,2-propanediol (Feed ratio = 0.8, Recycle ratio = 0.1), (**c**) recycle ratio-residence time on conversion of 1,2-propanediol (Temperature = 300 °C, Recycle ratio = 0.1).

Figure 7a shows the two and three-dimensional response surface curves to illustrate the simultaneous effects of temperature and feed ratio on the conversion of 1,2-propanediol. It is evident that increasing temperature improves the conversion, however; the effect of feed ratio on the conversion is negligible. effects of temperature and residence time on the conversion of 1,2-propanediol. It is seen that increasing temperature provides heat of reaction and improves the conversion. In low temperatures, the reaction doesn’t take place and variation of residence time can’t improve the conversion; however; in higher temperatures, increasing the residence time enhances the conversion of 1,2-propanediol. Figure 7b and c depict the two and three-dimensional response surface curves for the simultaneous effects of temperature-residence time and recycle ratio-residence time on the conversion of 1,2-propanediol, respectively. It is seen that increasing temperature provides heat of reaction and improves the conversion. In low temperatures, the reaction doesn’t take place and variation of residence time can’t improve the conversion; however; in higher temperatures, increasing the residence time enhances the conversion of 1,2-propanediol. It is also shown that increasing the residence time has positive effects on 1,2-propanediol conversion, but variation of recycle ratio has a mild effect on conversion of 1,2-propanediol. In addition; increasing both of them directly affects the cost of the process.

### Optimization of PC production process

For process optimization, effective parameters, their individual, square, and binary interactions on the response (1,2-propanediol conversion) have been studied with constraints presented in Table S4. The constraints have been determined according to process permissible conditions. According to Table S4, the residence time of 12.61 h, temperature of 295 °C, feed ratio of 0.8, and recycle ratio of 0.1 has been selected as an optimum point. The related conversion is 59.6%. According to Eq. (1), one mole of CO_2_ is consumed for the production of each mole of PC. Since the reaction takes place at a relatively low temperature, if the heat required for the reaction can be supplied using heat of other streams, more CO_2_ will not be produced due to combustion, and the net amount of consumed CO_2_ will be the same as the amount of produced PC. Otherwise, the amount of CO_2_ produced during combustion should also be considered.

## Conclusion

PC, a green solvent and an additive to fuel can be produced using 1,2-propanediol, an alcohol, and carbon dioxide, a major greenhouse gas. In this paper, the PC production process by1,2-propanediol and carbon dioxide has been studied, simulated, and optimized. The effective parameters of temperature, residence time, feed ratio, and recycle ratio have been investigated and their interactions on PC production have been evaluated. Temperature has been studied in the range of 75 to 400 °C and shown that increasing the temperature has a significant effect on the 1,2-propanediol conversion. Increasing the recycle ratio decreases the PC production rate by reducing the reactants' residence time in the constant reactor volume and increasing the residence time improves the conversion of the reactor. Based on the optimization of the process by RSM, the optimum point has been presented as the residence time of 12.61 h, temperature of 295 °C, feed ratio of 0.8, and recycle ratio of 0.1 which results in conversion of 59.6%.

### Supplementary Information

Supplementary Figure S1.

Supplementary Figure S2.

Supplementary Table S1.

Supplementary Table S2.

Supplementary Table S3.

Supplementary Table S4.

## Data Availability

All data generated or analysed during this study are included in this published article [and its supplementary information files].
