# Supplementary figures and images for: Carbon dioxide utilization in propylene carbonate production process
Source: Sci Rep. 2024 Jun 22;14:14422. doi: 10.1038/s41598-024-65115-z (PMC11193729; doi:10.1038/s41598-024-65115-z)

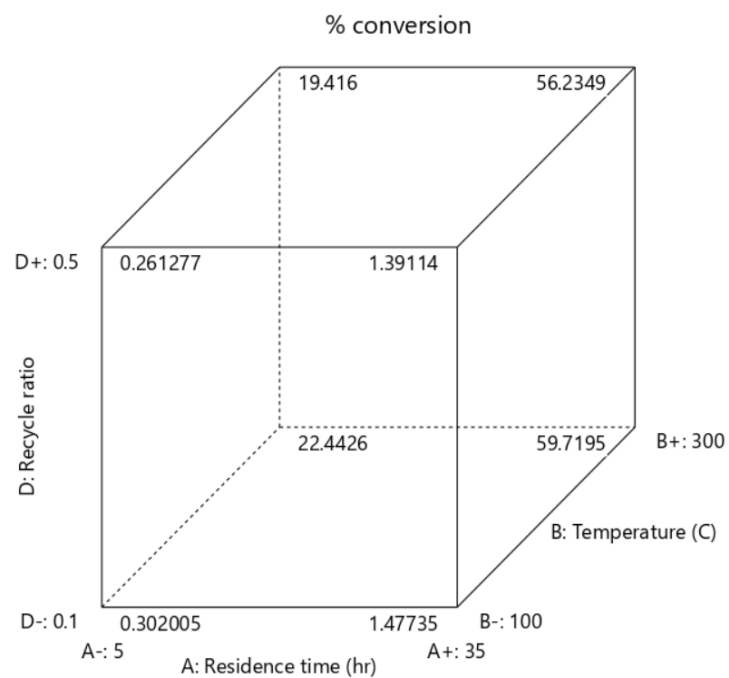

Figure S1: Box Behnken Design for propylene carbonate production (recycle ratio= 0.8)

Supplement: Supplementary file 1 — Supplementary Figure S1. [file 41598_2024_65115_MOESM1_ESM.pdf]

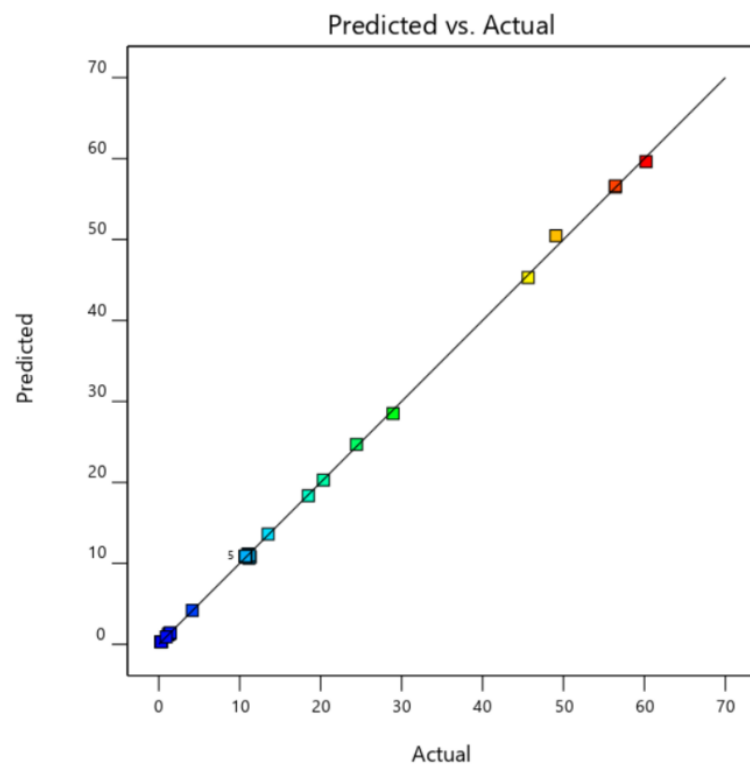

Figure S2: Predicted values of 1,2-propanediol conversion versus simulation results

Supplement: Supplementary file 2 — Supplementary Figure S2. [file 41598_2024_65115_MOESM2_ESM.pdf]
