# Supplementary material for: Carbon dioxide utilization in propylene carbonate production process
Source: Sci Rep. 2024 Jun 22;14:14422. doi: 10.1038/s41598-024-65115-z (PMC11193729; doi:10.1038/s41598-024-65115-z)
Supplement: Supplementary file 3 — Supplementary Table S1. [file 41598_2024_65115_MOESM3_ESM.docx]

Table S1: Kinetic parameters of synthesis of propylene carbonate [14].

| Coefficient and activation energy | Value |
| --- | --- |
| $k_{A1}$ | 1.491 |
| $k_{A2}$ | 9.751 |
| $k_{A4}$ | 0.048 |
| $k_{A5}$ | 1.794 |
| Ea (kJ/mol) | 77.2 |
