# Supplementary material for: Carbon dioxide utilization in propylene carbonate production process
Source: Sci Rep. 2024 Jun 22;14:14422. doi: 10.1038/s41598-024-65115-z (PMC11193729; doi:10.1038/s41598-024-65115-z)
Supplement: Supplementary file 4 — Supplementary Table S2. [file 41598_2024_65115_MOESM4_ESM.docx]

Table S2: Response surface values for PC production process

|  |  | Factor 1 | Factor 2 | Factor 3 | Factor 4 | Response 1 |
| --- | --- | --- | --- | --- | --- | --- |
| Std | Run | A: Resistance Time (hr) | B: Temperature (°C) | C: Feed Ratio (-) | D: Recycle Ratio (-) | Conversion (%) |
| 1 | 23 | 2 | 100 | 0.6 | 0.3 | 0.3 |
| 2 | 28 | 16 | 100 | 0.6 | 0.3 | 1.3 |
| 3 | 19 | 2 | 300 | 0.6 | 0.3 | 20.3 |
| 4 | 13 | 16 | 300 | 0.6 | 0.3 | 25.6 |
| 5 | 11 | 9 | 200 | 0.4 | 0.1 | 0.3 |
| 6 | 15 | 9 | 200 | 0.8 | 0.1 | 1.3 |
| 7 | 25 | 9 | 200 | 0.4 | 0.5 | 28.9 |
| 8 | 20 | 9 | 200 | 0.8 | 0.5 | 60.2 |
| 9 | 6 | 2 | 200 | 0.6 | 0.1 | 0.31 |
| 10 | 3 | 16 | 200 | 0.6 | 0.1 | 1.2 |
| 11 | 27 | 2 | 200 | 0.6 | 0.5 | 24.4 |
| 12 | 7 | 16 | 200 | 0.6 | 0.5 | 56.4 |
| 13 | 8 | 9 | 100 | 0.4 | 0.3 | 0.3 |
| 14 | 1 | 9 | 300 | 0.4 | 0.3 | 1.4 |
| 15 | 24 | 9 | 100 | 0.8 | 0.3 | 18.4 |
| 16 | 31 | 9 | 300 | 0.8 | 0.3 | 56.4 |
| 17 | 12 | 2 | 200 | 0.4 | 0.3 | 4.1 |
| 18 | 9 | 16 | 200 | 0.4 | 0.3 | 13.5 |
| 19 | 14 | 2 | 200 | 0.8 | 0.3 | 0.9 |
| 20 | 17 | 16 | 200 | 0.8 | 0.3 | 49.11 |
| 21 | 29 | 9 | 100 | 0.6 | 0.1 | 11.1 |
| 22 | 4 | 9 | 300 | 0.6 | 0.1 | 11.02 |
| 23 | 5 | 9 | 100 | 0.6 | 0.5 | 11.03 |
| 24 | 21 | 9 | 300 | 0.6 | 0.5 | 11.3 |
| 25 | 16 | 9 | 200 | 0.6 | 0.3 | 10.7 |
| 26 | 26 | 9 | 200 | 0.6 | 0.3 | 11.2 |
| 27 | 10 | 9 | 200 | 0.6 | 0.3 | 10.7 |
| 28 | 30 | 9 | 200 | 0.6 | 0.3 | 10.7 |
| 29 | 2 | 9 | 200 | 0.6 | 0.3 | 10.7 |
| 30 | 18 | 9 | 200 | 0.6 | 0.3 | 10.7 |
| 31 | 22 | 9 | 200 | 0.6 | 0.3 | 10.7 |
