# Supplementary material for: Carbon dioxide utilization in propylene carbonate production process
Source: Sci Rep. 2024 Jun 22;14:14422. doi: 10.1038/s41598-024-65115-z (PMC11193729; doi:10.1038/s41598-024-65115-z)
Supplement: Supplementary file 5 — Supplementary Table S3. [file 41598_2024_65115_MOESM5_ESM.docx]

Table S3: Analyses Of Variance (ANOVA)

| 0 | Sum of Squares | df | Mean Square | F-value | P-value |  |
| --- | --- | --- | --- | --- | --- | --- |
| **Model** | 84.01 | 15 | 5.60 | 10323.79 | < 0.0001 | significant |
| **A-Resistance Time** | 6.25 | 1 | 6.25 | 11515.82 | < 0.0001 |  |
| **B-Temp** | 72.58 | 1 | 72.58 | 1.338E+05 | < 0.0001 |  |
| **C-Feed Ratio** | 0.0106 | 1 | 0.0106 | 19.57 | < 0.0005 |  |
| **D-Recycle Ratio** | 0.0016 | 1 | 0.0016 | 1.88 | 0.1101 |  |
| **E-Pressure** | 0.0019 | 1 | 0.0019 | 3.43 | 0.0839 |  |
| **AB** | 0.3707 | 1 | 0.3707 | 683.34 | < 0.0001 |  |
| **AC** | 0.0136 | 1 | 0.0136 | 25.13 | 0.0002 |  |
| **AD** | 0.0072 | 1 | 0.0072 | 13.24 | 0.0024 |  |
| **AE** | 0.0822 | 1 | 0.0822 | 151.48 | < 0.0001 |  |
| **BC** | 0.0040 | 1 | 0.0040 | 7.41 | 0.0158 |  |
| **BE** | 0.0200 | 1 | 0.0200 | 36.90 | < 0.0001 |  |
| **CD** | 0.0281 | 1 | 0.0281 | 51.83 | < 0.0001 |  |
| **CE** | 0.0148 | 1 | 0.0148 | 27.20 | < 0.0001 |  |
| **A²** | 0.4628 | 1 | 0.4628 | 853.09 | < 0.0001 |  |
| **B²** | 0.7816 | 1 | 0.7816 | 1440.76 | < 0.0001 |  |
| **Residual** | 0.0005 | 15 | 0.0005 |  |  |  |
| **R²** | 0.9999 |  |  |  |  |  |
| **Adjusted R²** | 0.9998 |  |  |  |  |  |
| **Predicted R²** | 0.9995 |  |  |  |  |  |
| **Adeq Precision** | 317.6059 |  |  |  |  |  |
