# Supplementary material for: Carbon dioxide utilization in propylene carbonate production process
Source: Sci Rep. 2024 Jun 22;14:14422. doi: 10.1038/s41598-024-65115-z (PMC11193729; doi:10.1038/s41598-024-65115-z)
Supplement: Supplementary file 6 — Supplementary Table S4. [file 41598_2024_65115_MOESM6_ESM.docx]

Table S4- Applied constrains for independent variables

| Name | Goal | Lower Limit | Upper Limit | Lower Weight | Upper Weight | Importance |
| --- | --- | --- | --- | --- | --- | --- |
| A: Resistance Time | is in range | 2 | 16 | 1 | 1 | 3 |
| B: Temp | is in range | 100 | 300 | 1 | 1 | 3 |
| C: Feed Ratio | is in range | 0.4 | 0.8 | 1 | 1 | 3 |
| D: Recycle Ration | is in range | 0.1 | 0.5 | 1 | 1 | 3 |
| E: Pressure | is in range | 1000 | 9000 | 1 | 1 | 3 |
